# Clinical Significance and Diagnostic Value of Pain Extent Extracted from Pain Drawings: A Scoping Review

**DOI:** 10.3390/diagnostics10080604

**Published:** 2020-08-18

**Authors:** Marco Barbero, Marcos J. Navarro-Santana, María Palacios-Ceña, Ricardo Ortega-Santiago, Corrado Cescon, Deborah Falla, César Fernández-de-las-Peñas

**Affiliations:** 1 Rehabilitation Research Laboratory 2rLab, Department of Business Economics, Health and Social Care, University of Applied Sciences and Arts of Southern Switzerland, 6928 Manno, Switzerland; marco.barbero@supsi.ch (M.B.); corrado.cescon@supsi.ch (C.C.); 2 Department of Radiology, Rehabilitation and Physiotherapy, Universidad Complutense de Madrid, 28040 Madrid, Spain; marcosjose.navarrosantana@gmail.com; 3 Rehabilitación San Fernando, 28830 Madrid, Spain; 4 Department of Physical Therapy, Occupational Therapy, Physical Medicine and Rehabilitation, Universidad Rey Juan Carlos, 28922 Alcorcón, Spain; maria.palacios@urjc.es (M.P.-C.); ricardo.ortega@urjc.es (R.O.-S.); 5 Cátedra Institucional en Docencia, Clínica e Investigación en Fisioterapia: Terapia Manual, Punción Seca y Ejercicio Terapéutico, Universidad Rey Juan Carlos, 28922 Alcorcón, Spain; 6 Centre of Precision Rehabilitation for Spinal Pain (CPR Spine), School of Sport, Exercise and Rehabilitation Sciences, College of Life and Environmental Sciences, University of Birmingham, Birmingham B15 2TT, UK; d.falla@bham.ac.uk

**Keywords:** pain drawing, pain extent, patient-reported outcome measures, chronic pain

## Abstract

The current scoping review aimed to map current literature investigating the relationship between pain extent extracted from pain drawings with clinical, psychological, and psycho-physiological patient-reported outcome measures in people with pain. Electronic databases were searched for cross-sectional cohort studies that collected pain drawings using digital technology or a pen-on-paper approach and assessed for correlations between pain extent and clinical, psychological or psycho-physical outcomes. Data were extracted by two different reviewers. The methodological quality of studies was assessed using the Newcastle–Ottawa Quality Assessment Scale. Mapping of the results included: 1, description of included studies; 2, summary of results; and 3, identification of gaps in the existing literature. Eleven cross-sectional cohort studies were included. The pain disorders considered were heterogeneous, ranging from musculoskeletal to neuropathic conditions, and from localized to generalized pain conditions. All studies included pain and/or pain-related disability as clinical outcomes. Psychological outcomes included depression and anxiety, kinesiophobia and catastrophism. Psycho-physical measures included pressure or thermal pain thresholds. Ten studies were considered of high methodological quality. There was heterogeneity in the associations between pain extent and patient-reported outcome measures depending on the pain condition. This scoping review found that pain extent is associated with patient-reported outcome measures more so in patients presenting with musculoskeletal pain, e.g., neck pain or osteoarthritis, rather than for those with neuropathic pain or headache.

## 1. Introduction

A pain drawing (PD) is a self-report measure that helps patients to communicate the location and extent of their symptom. For a comprehensive patient evaluation, clinicians usually administer PDs together with other patient-reported outcome measures, for example, questionnaires capturing physical function or quality of life. Harold Palmer in 1949 proposed PDs as a tool to visualize the patient’s pain experience and specifically to support clinicians to differentially diagnose between “psychological pain” and “organic pain” [1]. Pain maps, pain drawings or pain charts are different terms used to describe a similar procedure which is to obtain a topographical description of the patient’s pain symptoms using a body chart (i.e., stereotypical image of the body) [2]. Although PDs are mainly used to evaluate the spatial distribution of pain, they have also been used to express emotional responses [3]. In fact, a combination of PDs and qualitative or quantitative descriptors have been proposed for a multidimensional evaluation of pain experience, e.g., McGill Pain Questionnaire [4]. The information obtained from the analysis of PDs can be used to complete the diagnostic history taking or to enhance the documentation of provocative tests. When used longitudinally, PDs can provide evidence of spontaneous regression or progression of the painful condition or can be used to evaluate the efficacy of treatment. 

The classic pen-on-paper approach or more modern acquisition using a digital tablet can be used to obtain PDs which are then assessed by visual inspection (i.e., qualitative analysis) or by digitalization of the PD (i.e., quantitative analysis). The latter is an objective and reliable method to quantify pain extent, defined as the area marked in the body chart boundaries [5,6,7].

The digital transformation in healthcare and the easy accessibility of technologies such as PCs, tablets and smartphones, have created many opportunities for the use of digital PD. A recent review reporting methodological milestones in PD acquisition and analysis has confirmed the need to focus on digital technology by investigating innovative methods to calculate PD-derived measures [8]. In the case of large datasets, a diagnostic potential has been attributed to pain pattern analysis by advanced statistical methods or artificial neural networks [9]. 

**Figure 1:**
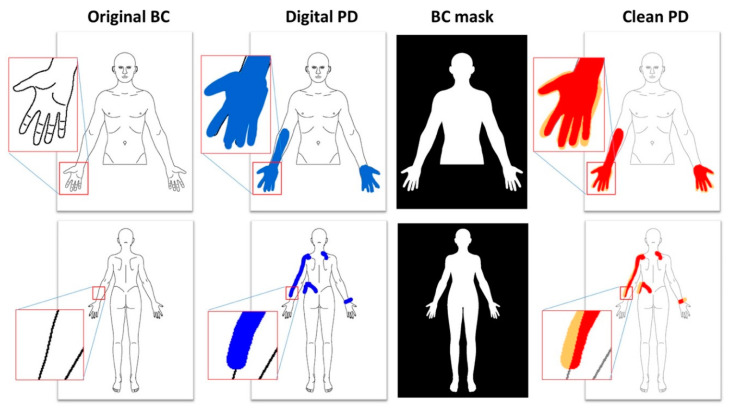
Examples of identification of pain area from digital pain drawings. The original body chart is colored by the patient with a blue digital marker, then a mask is applied in order to remove the colored pixels outside the body chart area. The pixels of the clean pain draw (represented in red) are counted to obtain the pain area. The pixels outside the body chart area (represented in orange) are discarded.

**Figure 2:**
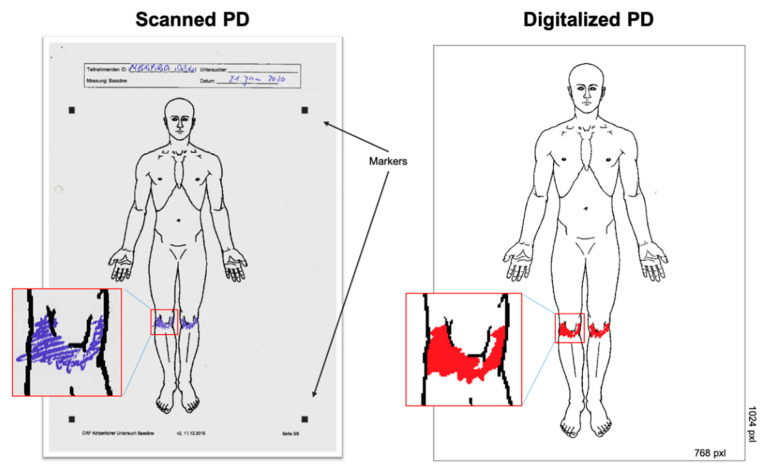
Examples of identification of pain area from paper body chart. The body chart is printed on A4 paper with black squares (markers) and additional information according to the protocol. The patient is asked to color the painful areas with a blue or red pen, then the pain drawing is scanned, scaled and aligned using the black markers as reference. The digital pain drawing is then analyzed as described in Figure 1.

Software-based procedures for obtaining and quantifying the extent and the location of pain have been developed i.e., those generated by a virtual colored marker on the screen of a tablet which avoids potential subjective influence from an assessor. The colored pixels inside the body chart are counted to determine the pain extent (Figure 1), while the pixels outside the borders of the body chart are discarded. In the case of paper PDs, the PD is scanned with a color scanner, then the body chart is rotated and scaled to match the size and orientation of the original body chart (this procedure is necessary as each scanner has a different resolution in terms of pixels per inch, and the scanned image may have a few degrees of misalignment). When the PD is correctly digitalized, scaled and aligned, in order to have the same dimensions as the original body chart (usually 1024 × 768 pixels), the number of colored pixels inside the chart is determined in order to obtain the pain extent (Figure 2). The colored pixels belonging to the PD can be distinguished by the black or grey pixels of the chart contours using a simple threshold based on the difference between two layers of the R.G.B composite image. When a PD is colored with a red marker, the [R.G.B] vector of each pixel will have higher values in the first component and lower values on the other two components (ideally [255-0-0]), while each point of the chart or the background will have equal values in the [R.G.B] vector. This approach to determine pain extent has shown good to excellent reliability for patient’s self-report of pain location in clinical pain populations [10] and for assessing pain responses to a provocative test in an asymptomatic population [11].

Over the last few decades, a considerable amount of clinical investigations had examined pain extent. However, the clinical significance or the diagnostic utility of pain extent, determined digitally from the PD, is not fully understood. Scoping reviews are an ideal tool to map current and emerging scientific evidence of a topic by providing an indication for future research in those circumstances where systematic reviews are unable to meet the necessary objectives of knowledge users [12]. Systematic reviews usually address feasibility, appropriateness, meaningfulness or effectiveness of a therapeutic intervention or diagnostic procedure to better guide decisions in clinical practice, whereas scoping reviews are used to identify, report, and discuss the available evidence on a specific topic/concept, an appraisal procedure usually referred as “evidence mapping” [12]. Its findings are highly relevant as they inform practice, reveal research gaps or determine the value for undertaking a full systematic review [12]. Accordingly, the current scoping review aimed to map the existing literature investigating the relationship between pain extent, extracted from PDs, with clinical, psychological and psycho-physiological patient-reported outcome measures in people with pain.

## 2. Methods

A scoping review design was chosen to lead a broad overview of the available research on pain extent, where heterogeneity of methods and populations was admitted/comprised [13]. The methodological framework followed five stages suggested by Arksey and O’Malley [14]: 1, Identify the research question; 2, Identify relevant studies; 3, Study selection; 4, Chart the data; 5, Collate, summarize and report the results. To ensure a transparent and accurate reporting structure, the Preferred Reporting Items for Systematic Reviews and Meta-Analyses Extension for Scoping Reviews (PRISMA-ScR) was adopted [15]. This scoping review was prospectively registered on the OSF database https://doi.org/10.17605/OSF.IO/UFCWH.

### 2.1. Identify the Research Question

The research question was defined in order to highlight the possible clinical significance and the diagnostic utility of pain extent obtained from the PD (either collected using the pen-on-paper approach or directly compiled using a digital tablet). The key question of the current scoping review was: what is the relationship between pain extent and clinical, psychological and psycho-physiological patient-reported outcome measures in individuals with pain?

### 2.2. Identify Relevant Studies 

Electronic literature searches were conducted on the following databases from January 2000 to 1 July 2020: PubMed, MEDLINE, EMBASE, AMED, CINAHL, EBSCO and SCOPUS. We also screened the reference lists of the papers identified in the databases. Key journals were also hand-searched to identify articles that have been missed in database and reference list searches. A database literature search was conducted with the assistance of an experienced health science librarian. Searches were limited to human studies and English-language citations. To avoid missing any relevant study, the following terms were combined by using Boolean operators: “pain drawing”, “pain draw”, “pain extent”, “pain chart”, “pain area”, “pain map” AND “pain”, “related-disability” “function”, “mobility”, “quality of life”, “anxiety”, “depression”, “quantitative sensory testing”, “pain thresholds”. Table 1 details the search strategy for each database.

### 2.3. Study Selection

The eligibility criteria were defined a priori by authors and then finalized through a pilot title and abstract screen. We used the PCC mnemonic (Population, Concept and Context) to define the inclusion criteria [16]:

Population: Adults suffering from any painful condition.

Concept: Collection of PDs using digital technologies or the pen-on-paper approach with pain extent determined digitally (i.e., software calculated).

Context: Definition of potential correlation between pain extent and patient-reported outcome measures, and/or psycho-physical outcomes, e.g., quantitative sensory testing (QST).

This scoping review considered primary research including observational studies (i.e., case control or cohort studies). Two authors reviewed publications identified in the search databases by reviewing the title and abstract of the text. A full-text read of potential eligible studies was conducted. 

Discrepancies in the reviewers’ responses at any stage of the screening were resolved by asking a third author, if necessary. 

### 2.4. Chart Data

Data extraction in scoping reviews is conducted with a “data charting form” in which a descriptive summary of the results is generated [14]. A data charting form was developed for this scoping review to identify the variables that correspond with the research question. Data were extracted independently by two authors using a data charting form including authors, year of publication, population, sample size, diagnosis, pain draw, clinical or psychological patient-reported outcome measures and psycho-physical outcomes [13]. Both authors had to achieve consensus on each item on the data charting form. If disagreement occurred, a third author participated in the decision to reach resolution.

### 2.5. Data Mapping

After data extraction, we mapped the literature thematically, according to the following topics: 1, description of the identified and included studies; 2, summary of the results; and, 3, identification of gaps in the existing literature.

### 2.6. Methodological Quality

The methodological quality of the studies was independently assessed by two authors using the Newcastle–Ottawa Scale, a star rating system (a maximum nine of stars) that evaluates the risk of bias of case-control and cohort studies [17]. This scale, when applied to cohort studies, includes three main sections: case selection, comparability, and exposure. 

Case selection includes the following four items: representativeness of the cohort, selection of non-exposed cohort, ascertainment of exposure (case definition), and outcome of interest nor present at start. Comparability evaluates the analysis of comparison (e.g., controlled for age, gender, or other factors) between groups (exposed and non-exposed). Exposure includes three items: assessment of outcome, long enough follow-up period, and adequate follow-up. In longitudinal cohort studies, a maximum of 9 stars can be awarded. Studies scoring ≥ 7 are considered of good quality, those scoring 5 or 6 are of fair quality and studies scoring 0–4 are of poor quality [18]. In cross-sectional cohort studies, a maximum of 3 stars can be awarded. Studies scoring 3 are considered of good quality, those scoring 2 are of fair quality and studies scoring 1 are of poor quality. Risk of bias/methodological quality of the included studies was again determined by two authors and the differences, if existed, were discussed. In the case of disagreement between the two authors, a third researcher arbitrated a consensus decision. 

## 3. Results

### 3.1. Study Selection

**Figure 3:**
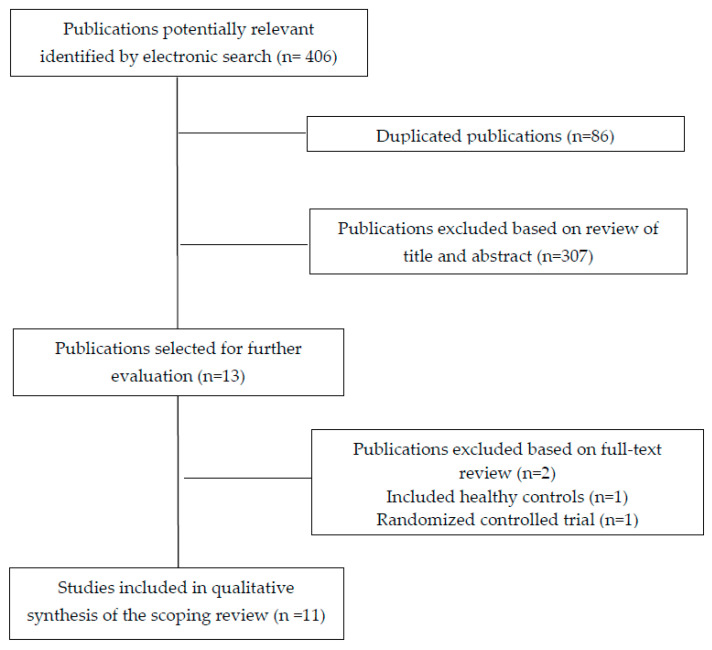
PRISMA Extension for Scoping Reviews (PRISMA-ScR) flow diagram.

The electronic searches identified 406 potential studies for review. After removing duplicates and those not related to the topic, 13 studies remained for full-text analysis. From the thirteen studies that were examined, two were excluded, the first one because it included asymptomatic individuals [11], and the second one because it was a randomized controlled clinical trial [19]. A total of eleven cross-sectional cohort studies [10,20,21,22,23,24,25,26,27,28,29] were included in the final literature data mapping (Figure 3).

### 3.2. Study Characteristic

Eleven cross-sectional cohort studies analyzed the association of pain extent with clinical outcomes related to pain and related-disability, psychological patient-reported outcome measures (e.g., depression, anxiety, kinesiophobia, or catastrophism), and psycho-physical outcomes (e.g., pressure or thermal pain thresholds). 

The characteristics of the populations of the included studies are shown in Table 2. The total sample included 1114 patients (372 men, 742 women) with samples ranging from 30 to 205 subjects. Five studies included individuals with musculoskeletal pain (e.g., neck pain, low back pain); two included patients with widespread pain conditions with potential neuropathic pain component (i.e., whiplash-associated disorders or fibromyalgia); two included patients with primary headaches (i.e., tension-type headache or migraine); and the last one included patients with neuropathic pain (e.g., carpal tunnel syndrome). The pain conditions investigated in the studies were heterogeneous and included patients with mechanical neck pain (*n* = 3), low back pain (*n* = 1), whiplash-associated disorders (*n* = 1), fibromyalgia syndrome (*n* = 1), carpal tunnel syndrome (*n* = 1), tension-type headache (*n* = 1), migraine (*n* = 1), knee osteoarthritis (*n* = 1), hip osteoarthritis (*n* = 1), or work-related neck pain (*n* = 1). 

All studies used digital software-based computation for calculating pain extent with six of them collecting data with digitalized PDs on a tablet [10,20,21,24,27,28] and five using a pen-to-paper approach [22,23,25,26,29]. Across all studies, the pain extent ranged from 6.7% to 22% (mean ± SD: 12.1% ± 5.3%), and in all cases its computation was software-based as per the inclusion criteria. In addition, all studies included pain-related outcomes in the following domains: intensity, duration, pain-related disability and function. Psychological patient-reported outcome measures included depression (*n* = 6) [21,22,25,27,28,29], anxiety (*n* = 5) [21,22,25,27,28], kinesiophobia (*n* = 4) [20,21,28,29] and catastrophism (*n* = 3) [21,27,28]. Psycho-physical measures mostly included pressure and thermal thresholds. Pressure pain thresholds (PPTs) were assessed in seven studies in symptomatic and distant pain-free areas for assessing widespread pressure pain sensitivity [20,21,22,23,25,26,28], whereas thermal pain thresholds (cold pain, CPT and heat pain, HPT) were assessed in four studies [23,25,26,28]. Other measures, e.g., central sensitization inventory or conditioning pain modulation were also assessed, but only in one study [20] (Table 2). 

### 3.3. Methodological Quality

The methodological quality score was 3 stars (maximum score in cross-sectional cohort designs) in ten studies [10,20,21,22,23,24,25,26,28,29] and 2 stars in the last one [27]. No disagreement between authors was observed. Table 3 presents the Newcastle–Ottawa Scale for each cross-sectional cohort study and a summary of every item.

### 3.4. Summarizing Findings

There was heterogeneity in the results between the association between pain extent and clinical, psychological and psycho-physical outcomes depending on the pain condition. The findings revealed that larger pain extent was significantly associated with:

1. higher pain intensity in individuals with mechanical [10,29], whiplash-associated [21] or work-related [24] neck pain, and in those with fibromyalgia syndrome [23];
2. greater disability in mechanical, traumatic or work-related neck pain [10,21,24,29] and knee osteoarthritis [20];
3. higher pressure pain hyperalgesia (lower pressure pain threshold) in knee [20] and hip [28] osteoarthritis;
4. higher depressive symptoms in mechanical [10,29] and whiplash-associated [21] neck pain.

Although some isolated associations were also seen, in general, no relationship was observed between pain extent and clinical, psychological or psycho-physical outcomes in women with chronic tension-type headache [22], episodic migraine [25] or carpal tunnel syndrome [26].

## 4. Discussion

This is the first scoping review investigating the relationship between pain extent extracted from PDs with clinical or psychological patient-reported outcome measures, and psycho-physiological outcomes in patients with pain. 

### 4.1. Literature Mapping

It is accepted that an expanded distribution of pain represents a clinical sign of central sensitization [30]. Central sensitization was operationally defined by Woolf as an amplification of neural signaling within the central nervous system that elicits pain hypersensitivity [31]. This term is a broad concept integrating several complex pathophysiological mechanisms such as spinal cord sensitization, impaired functioning of the descending inhibitory mechanisms, (over) activation of descending pain facilitatory pathways, increased temporal summation (wind-up) and alteration of sensory processing in the brain [32]. Greater pain extent can be attributed to the development of spreading pain sensitization mechanisms at the spinal cord, particularly the activation of quiescent neurons in the dorsal horn [30]. Therefore, a precise quantification of the symptomatic area helps to better describe, and especially to objectify, the pain experience in people suffering from chronic pain. This was the main reason for the development of a software for the automated computation of pain extent. It constitutes a meaningful advancement when compared to tools like the Widespread Index [31], especially since it can provide an accurate topographic distribution of the patient’s symptoms.

This scoping review found that pain extent was associated with clinical outcomes, i.e., pain intensity and related disability, in painful conditions of musculoskeletal origin such as neck pain, osteoarthritis, and fibromyalgia syndrome [10,20,21,23,24,29], but not in conditions of non-musculoskeletal origin such as carpal tunnel syndrome (neuropathic pain) or headache (conditions associated to a deficient regulation of excitatory-inhibitory balance and activation of the trigemino-vascular system). Lluch-Girbés et al. found no association between more expanded distributions of pain (pain extent) and self-reported neuropathic pain scores in individuals presenting with knee osteoarthritis [20]. It is likely that musculoskeletal pain has a different impact on self-reported perception of pain than neuropathic nociception. This may be related to the quality of subjective symptoms characterizing neuropathic pain (i.e., burning, shooting, flashing). Nevertheless, it should be noted that only a small (5.4–14.8%) percentage of people with osteoarthritis suffer from neuropathic pain [20,33].

The association between pain extent and psychological patient-reported outcome measures was small since it was only found in patients with mechanical neck pain [10,29] and whiplash-associated disorders [21]. Overall, the current findings should be considered in line with the two previous systematic reviews which did not report an association between pain extent and psychological status nor a diagnostic utility for PDs [3,34]. Nevertheless, it is important to note that the cited reviews, in their synthesis of the evidence, did not focus on the value of pain extent extracted from PDs. It is important to consider that pain extent covers one aspect of the pain spectrum whereas psychological patient-reported outcome measures cover other (more cognitive) components. Enlarged areas of pain may be perceived by the patient as more life-threatening and, therefore, this emotional state could lead to worse self-perceived burden or higher related disability. It has recently been proposed that examination of patients with pain should cover multiple domains of the pain experience including, among others, the extension, location and the distribution of pain [35]. The use of a software-based computation for calculating pain extent will help to achieve this objective.

We also observed that large pain extent was associated with higher widespread pressure pain hyperalgesia in individuals with knee [20] or hip [28] osteoarthritis. Both outcomes, i.e., enlarged areas of pain and widespread pressure pain hyperalgesia, are clinical manifestations of central sensitization, which is found in several musculoskeletal pain conditions supporting this relationship. However, this association was not reported in other musculoskeletal pain conditions such as whiplash-associated disorders [21] or fibromyalgia syndrome [23]. It is plausible that different drivers could lead to different associations between sensitization outcomes in different musculoskeletal pain disorders. This hypothesis agrees with recent theories supporting that, although central sensitization is present in both musculoskeletal and neuropathic pain conditions, its assessment and evaluation could be different since their manifestations can vary from pain condition to pain condition [36]. In addition, it can be also related to the fact that whiplash-associated disorders and fibromyalgia also exhibit a neuropathic pain component in their underlying mechanisms.

### 4.2. Limitations of Current Literature and Implications for Research

The pain extent experienced by a patient has potential implications for clinical practice. First, digital assessment and calculation of pain extent is an objective measure not affected by subjective interpretation by the assessor. Therefore, this assessment could be used to monitor clinical evolution of symptoms during the course of the disease (natural progression of the condition), after a treatment intervention (effect of a therapeutic approach) or as a potential prognostic factor for clinical outcomes. Nevertheless, it should be recognized that patient extent and PDs cannot be used alone to determine the patient’s diagnosis, since several pain conditions exhibit overlapping symptom locations. This is also highly important considering that pain extent represents a quantitative, not qualitative, experience of pain. In fact, although digital assessment of the pain extent is objectively calculated, it should be considered that this calculation is based on the subjective experience of a patient. Therefore, when patients present with different symptoms (e.g., deep pain, sharp pain, electrical pain, burning pain) in the same area, it would be difficult to register the extent of symptoms according to each sensation reported by the patient. The use of symbols (e.g., crosses, circles, hatchings), or colors (e.g., red, black, blue), associated with each type of symptom (burning, electrical) or sensory disturbance (hyperalgesia, allodynia, hypoesthesia) [37] could help to determine the extent of symptoms depending on each sensory (or motor) symptom.

Additionally, it should be also noted that most studies included in this scoping review included static outcomes of sensitization (pressure or thermal pain thresholds). Since central sensitization is a dynamic process, we do not know if pain extent is associated with dynamic outcomes of central sensitization e.g., wind-up, spatial or temporal summation, or conditioned pain modulation. Based on the available literature, we cannot exclude a potential association between pain extent and other outcomes of sensitization.

### 4.3. Strengths and Limitations of the Review

The results from the current scoping review should be analyzed according to its potential strengths and limitations. Strengths of this scoping review include a comprehensive literature search, methodological rigor, data extraction and the inclusion of studies (cross-sectional cohort studies) of high methodological quality. However, some potential limitations are also present. First, the number of studies initially identified was up to 406, but only a relatively small number (*n* = 11) were included. The most important issue was the heterogeneity in the conditions included. Second, most studies are from the same research team, however, it should be noted that the included studies were conducted in different European countries. Third, all studies used a cross-sectional cohort design, which limits the clinical application or the predictive relevance of pain extent calculation. As it has been previously commented, studies investigating the predictive value of pain extent during the natural course of pain condition or the prognostic value of pain extent in clinical treatment outcomes would help to further elucidate the clinical relevance of this assessment. 

## 5. Conclusions

This scoping review investigated the relationship between pain extent and clinical or psychological patient-reported outcome measures and psycho-physiological outcomes in individuals with pain. The results were heterogeneous in relation to the pain condition and the presence or not of associations between the outcomes. Based on the available data, pain extent is associated with patient-reported outcome measures more so in individuals suffering from musculoskeletal pain, e.g., neck pain or osteoarthritis, rather than with those presenting with neuropathic pain or headaches.

## Figures and Tables

**Table 1:** Database formulas during literature search.

**PubMed Search Formula** #1 “Pain Drawing” [Title/Abstract] OR “Pain Draw” [Title/Abstract] OR “Pain Extent” [Title/Abstract] OR “Pain Chart” [Title/Abstract] OR “Pain Area” [Title/Abstract] OR “Pain Map” [Title/Abstract] #2 “Pain” [Mesh] OR “Related-disability” [Title/Abstract] OR “Function” [Title/Abstract] OR “Mobility” [Title/Abstract] OR “Quality of Life” [Title/Abstract] OR “Anxiety” [Mesh] OR “Depression” [Mesh] OR “Quantitative Sensory Testing” [Title/Abstract] OR “Pain Thresholds” [Mesh] #3 #1 AND #2
**CINAHL/Medline (via EBSCO) Search Formula** #1 “Pain Drawing” OR “Pain Draw” OR “Pain Extent” OR “Pain Chart” OR “Pain Area” OR “Pain Map” #2 “Pain” OR “Related-disability” OR “Function” OR “Mobility” OR “Quality of Life” OR “Anxiety” OR “Depression” OR “Quantitative Sensory Testing” OR “Pain Thresholds” #3 #1 AND #2
**SCOPUS Search Formula** TITLE-ABS-KEY (“Pain Drawing” OR “Pain Draw” OR “Pain Extent” OR “Pain Chart” OR “Pain Area” OR “Pain Map”) AND TITLE-ABS-KEY (“Pain” OR “Related-disability” OR “Function” OR “Mobility” OR “Quality of Life” OR “Anxiety” OR “Depression” OR “Quantitative Sensory Testing” OR “Pain Thresholds”)
**WOS (EMBASE, AMED) Search Formula** (“Pain Drawing” OR “Pain Draw” OR “Pain Extent” OR “Pain Chart” OR “Pain Area” OR “Pain Map”) AND (“Pain” OR “Related-disability” OR “Function” OR “Mobility” OR “Quality of Life” OR “Anxiety” OR “Depression” OR “Quantitative Sensory Testing” OR “Pain Thresholds”)

**Table 2:** Characteristics of included studies.

Author	Population	Sample (Men/Women)	Mean Age (Years)	Outcome	Results Summary
Barbero et al. [10]	Neck Pain Low Back Pain	56 (15/41) 51 (20/31)	50.3 (15.0) 48.5 (14.1)	Pain Extent Clinical outcomes: BMI, age, pain duration, pain (VAS, 0–100), function (NDI, or RMDQ) Psychological outcomes: K-10, MoCa	Significant correlations were observed between pain extent and pain intensity in both conditions, between pain extent and disability in neck pain
Lluch-Girbés et al. [20]	Knee Osteoarthritis	53 (19/34)	70.2 (7.4)	Pain Extent Clinical outcomes: Pain (NPRS, 0–10), function (WOMAC) Psychological outcomes: PCS, PVAQ, CPAQ, TSK Psycho-physical outcomes: PPTs, CPM, CSI, PD-Q	Significant positive correlations between pain extent with pain and stiffness subscales of WOMAC and CSI score were found. Significant negative correlations between pain extent and PPTs were found
Falla et al. [21]	Whiplash-associated disorders	205 (133/72)	40.1 (11.4)	Pain Extent Clinical outcomes: Pain (VAS, 0–100), function (NDI, 0–100), PDI, EQ-5D Psychological outcomes: TSK, PCS, HADS-A, HADS-D, SES Others: Effort-Reward Imbalance Scale	Pain extent was influenced by sex, insurance status and worse financial situation. Positive significant associations between pain extent with NDI, HADS-D and PDI were found. A negative significant association between pain extent and SES was found
Palacios-Ceña et al. [22]	Chronic Tension-Type Headache	99 (27/72)	47 (44–50)	Pain Extent Clinical outcomes: Age, headache intensity, duration and frequency, HDI-E, HDI-P Psychological outcomes: HADS-A, HADS-D, STAI-T, STAI-S. Psycho-physical outcomes: PPTs	Significant positive associations were found between pain extent with age and the burden of the headache (HDI-E and HDI-P)
Barbero et al. [23]	Fibromyalgia	30 (0/30)	52 (12) Median	Pain Extent Clinical outcomes: Age, pain duration, pain (NPRS, 0–10), function (FIQ), tender point count Psycho-physical outcomes: PPTs, HPTs, CPTs	Significant negative correlations were observed between pain extent with age and pain duration A significant positive association between pain extent and worst level of pain was found
Cruder et al. [24]	Musicians	158 (68/90)	22.4 (3.6)	Pain extent Clinical outcomes: BMI, practicing (hours), pain intensity (1–5), function (QD Score, and QD score optional module)	Significant positive correlations between pain extent with pain intensity, QD and QD optional module were found
Fernández-de-las-Peñas et al. [25]	Episodic Migraine	72 (0/72)	42 (10.22)	Pain extent Clinical outcomes: Age, migraine intensity, duration and frequency Psychological outcomes: HADS-A, HADS-D, STAI-T, STAI-S. Psycho-physical outcomes: PPTs	No significant associations between pain extent with any clinical, psychological or psycho-physical variables were observed
Fernández-de-las-Peñas et al. [26]	Carpal Tunnel Syndrome	140 (0/140)	47 (13.5) median	Pain extent Clinical outcomes: Age, pain duration, pain (NPRS, 0–10), function (BCTQ, 0–5) Psycho-physical outcomes: PPTs, HPTs, CPTs	A significative positive correlation between pain extent and CPT over carpal tunnel was observed
Ris et al. [29]	Neck pain Traumatic Nek Pain Non-traumatic Neck Pain	200 (75/125) 120 80	43.5 (11.4) 47.6 (11.4)	Pain Extent Clinical outcomes: Function (NDI), quality of life (SF36) Psychological outcomes: BDI-II, TSK Others: ROM, CCFT	Significative positive correlations between pain extent with NDI (all groups), BDI-II (all groups) and TSK (neck pain and nontraumatic pain groups) were seen Significative negative correlations between pain extent and muscle function (CCFT and CE) in neck pain and nontraumatic pain groups) were observed
Abichandani et al. [27]	Chronic Neck Pain	20 (0/20)	26 (2–32) Median	Pain Extent Recognition of pain drawing Clinical outcomes: age, pain duration, pain (NPRS, 0–10), NDI Psychological outcomes: PCS, DASS-42, MSPQ	A significative negative correlation was observed between recognition of pain drawing and MSPQ
Willet et al. [28]	Hip Osteoarthritis	30 (15/15)	61 (55.25–64) median	Pain extent Clinical outcomes: Age, function (Oxford Hip Score), pain (WPI), symptoms (FMS-SSS), PD-Q Psychological outcomes: DASS, TSK, PCS, CPAQ Psycho-physical outcomes: PTTs, CPTs, HPTs	Paint extent demonstrated significant association with WPI and PD-Q Pain extent was also associated with lower PPTs in the lower extremity, higher CPTs at the greater trochanter, reduced HPTs at the greater trochanter and reduced WDTs over the thenar eminence

BMI: Body Mass Index; BCTQ: Boston Carpal Tunnel Questionnaire; BDI-II Beck Depression Inventory; CCFT: Cranio-cervical flexion test; CE: Cervical Extension; CPAQ: Chronic Pain Acceptance Questionnaire; CPM: Conditioned Pain Modulation; CPT: Cold Pain Threshold; CSI: Central Sensitization Inventory; DASS-42: Depression Anxiety and Stress Scale; DASS: Depression, Anxiety, Stress 21 Scale; FIQ: Fibromyalgia Impact Questionnaire; FMS-SSS: Fibromyalgia Symptom Scale; HADS-A: Hospital Anxiety and Depression Scale (Anxiety subscale); HADS-D: Hospital Anxiety and Depression Scale (Depression subscale); HPT: Heat Pain Threshold; K-10: Kessler Psychological Distress Scale; MoCa: Montreal Cognitive Assessment; MSPA: Modified Somatic Perceptions Questionnaire; NDI: Neck Disability Index; NPRS: Numeric Pain Rating Scale; PCS: Pain Catastrophizing; PDI: Pain Disability Index; PD-Q: Pain DETECT questionnaire; PPT: Pressure Pain Threshold; PVAQ: Pain Vigilance and Awareness; QD: Quick DASH; RMDQ: Roland and Morris Disability Questionnaire; ROM: Range of motion; SES: Self-Efficacy Scale; SF-36-MCS: Short-Form 36, Standardized Mental Component Summary Score; SF-36-PCS: Short-Form 36, Standardized Physical Component Summary Score; TS: Temporal summation; TSK-11: 11-item Tampa Scale of Kinesiophobia; VAS: Visual Analogue Scale WOMAC: Western Ontario and McMaster Universities Arthritis Index; WPI: Widespread Pain Index.

**Table 3:** Newcastle–Ottawa Quality Assessment Scale. Quality appraisal for cohort studies.

	Selection				Comparability		Exposure			
Study	Representativeness of Exposed Cohort	Selection of Non-Exposed Cohort	Ascertainment of Exposure	Outcome of Interest nor Present at Start	Study Controls for Age/Gender	Study Controls for Additional Factor	Assessment of Outcome	Long Enough Follow-Up	Adequate Follow-Up	Score
Barbero et al. [10]	★		★				★			3/3
Lluch-Girbés et al. [20]	★		★				★			3/3
Falla et al. [21]	★		★				★			3/3
Palacios-Ceña et al. [22]	★		★				★			3/3
Barbero et al. [23]	★		★				★			3/3
Cruder et al. [24]	★		★				★			3/3
Fernández-de-las-Peñas et al. [25]	★		★				★			3/3
Fernández-de-las-Peñas et al. [26]	★		★				★			3/3
Ris et al. [29]	★		★				★			3/3
Abichandani et al. [27]			★				★			2/3
Willet et al. [28]	★		★				★			3/3

3/3 was considered a good quality cross-sectional cohort study; 2/3 was fair; 1/3 was considered poor quality.
